# Validating the effectiveness of Clinically Oriented Physiology Teaching (COPT) in undergraduate physiology curriculum

**DOI:** 10.1186/1472-6920-8-40

**Published:** 2008-07-24

**Authors:** Reem Abraham, Komattil Ramnarayan, Asha Kamath

**Affiliations:** 1 Melaka Manipal Medical College, Manipal, Karnataka, India

## Abstract

**Background:**

It has been proved that basic science knowledge learned in the context of a clinical case is actually better comprehended and more easily applied by medical students than basic science knowledge learned in isolation. The present study intended to validate the effectiveness of Clinically Oriented Physiology Teaching (COPT) in undergraduate medical curriculum at Melaka Manipal Medical College (Manipal Campus), Manipal, India.

**Methods:**

COPT was a teaching strategy wherein, students were taught physiology using cases and critical thinking questions. Three batches of undergraduate medical students (n = 434) served as the experimental groups to whom COPT was incorporated in the third block (teaching unit) of Physiology curriculum and one batch (n = 149) served as the control group to whom COPT was not incorporated. The experimental group of students were trained to answer clinically oriented questions whereas the control group of students were not trained. Both the group of students undertook a block exam which consisted of clinically oriented questions and recall questions, at the end of each block.

**Results:**

Comparison of pre-COPT and post-COPT essay exam scores of experimental group of students revealed that the post-COPT scores were significantly higher compared to the pre-COPT scores. Comparison of post-COPT essay exam scores of the experimental group and control group of students revealed that the experimental group of students performed better compared to the control group. Feedback from the students indicated that they preferred COPT to didactic lectures.

**Conclusion:**

The study supports the fact that assessment and teaching patterns should fall in line with each other as proved by the better performance of the experimental group of students compared to the control group. COPT was also found to be a useful adjunct to didactic lectures in teaching physiology.

## Background

From the early 1950s onwards, many medical schools have experimented with so-called innovative curricula, all of which have in one way or another achieved some form of integration of individual basic sciences (for instance, systems-based curricula), or of basic and clinical sciences (for instance, curricula based on problem-based learning). In particular, medical educators who have investigated the effects of problem-based learning have found benefits of basic science knowledge learned in a context of clinical problems [1,2]. There is also evidence that basic science knowledge learned in the context of a clinical case is actually better comprehended and more easily applied by medical students than basic science knowledge learned in isolation [3-5]. Medical educators who have investigated the effects of problem-based learning have found benefits of basic science knowledge learned in a context of clinical problems [1,2]. At the same time, PBL does not serve its purpose if students do not come prepared for it.

Educators agree that clinical reasoning is a central component of physician competence and objectives related to mastery of clinical reasoning skills appear in the documentation of most medical schools [6]. In most medical curricula, considerable attention has been given to the definition of the curriculum, to the organization of teaching and to the conduction of assessments. Little attention has been given to the impact of these activities on the way students learn. Taking the facts above into consideration, Clinically Oriented Physiology Teaching (COPT) was implemented to achieve two goals: first, to develop critical-thinking skills in undergraduate medical students to help them understand and apply the basic physiological concepts in clinical practice later and to improve their performance in clinically oriented questions in the examinations; second, to create an active learning environment so as to motivate the students to learn physiology. An earlier work on COPT by the authors had revealed that students' performance was better in an exam which was conducted after the incorporation of COPT, when compared to their performance in an exam conducted before the incorporation of COPT. Further in another study, the authors reported an increase in the deep approach and strategic approach and a mild decrease in surface approach by the same students after the implementation of COPT [7]. The present study attempted to empirically validate the effectiveness of COPT in undergraduate physiology curriculum.

## Methods

The undergraduate medical program at Melaka Manipal Medical College (MMMC) Manipal Campus, is a five-year, intense academic program. There are two admission intakes per year; one in March and another in September. Students are taught preclinical subjects in the first year. The first-year subjects include Anatomy, Physiology, and Biochemistry. This study was conducted at the department of Physiology, MMMC, Manipal Campus. The first-year curriculum is divided into four blocks as follows:

• Block 1: basic concepts, blood and nerve-muscle physiology

• Block 2: cardiovascular, respiratory, and gastrointestinal physiology

• Block 3: endocrine, reproductive, and renal physiology

• Block 4: central nervous system and special senses

At the end of each block, students undertook an examination which consisted of essay questions (Paper 1: out of 50) and multiple choice questions (Paper 2: out of 30). Paper 1 consisted of clinically oriented questions (Table 1) which accounted for 30–35% and direct questions which accounted for 15–20%. Three batches of first year MBBS (Bachelor of Medicine and Bachelor of Surgery) students (March 2003; n = 155; September 2003; n = 140 and March 2004; n = 139) were taken as the experimental groups and one batch (September 2004; n = 149) served as the control group for the study. COPT was incorporated along with the regular lectures in the third block to the three batches which served as the experimental group. COPT consisted of two components: I) clinical case studies ii) Critical Thinking Questions (CTQ). Critical Thinking Questions were questions which asked the physiological basis for some physiological concepts. Case studies and CTQ on particular topics were collected from books and from different websites. Those case studies and CTQ which were out of the learning objectives were omitted. Those which were selected were modified to match with the learning objectives. After teaching particular topics in each system, students were administered with the final version of CTQ and clinical case studies concerned with those topics. Students were asked to work on those questions. In the subsequent class, students were asked to present the answers. The misconceptions and doubts were clarified during the discussion. This was continued till the end of the block for the three batches of students.

**Table 1:** 

**Endocrinology**

1. Maria, a 40 year old woman presents to clinic with complaints of weakness, weight loss and heat intolerance. She had noticed increased appetite over the past few weeks and more frequent bowel movements over the same period. On examination her resting heart rate was found to be 85 beats/minute and the physician noticed slight protrusion of her eye balls and a swelling in the left anterior neck. Plasma TSH concentration was found to be low. She was prescribed propyl thiouracil by the physician.
a. Name the above endocrine disorder and justify your answer.
b. Is this a primary or secondary disorder? Why?
c. In the form of a flow chart describe the regulation of secretion of the hormone involved in the above case.
d. Mention the mechanism of action of propyl thiouracil.
e. Give the physiological basis for any one feature mentioned above
2. Give the physiological basis for the following:
a. Osteoporosis in Cushing's syndrome

**Reproductive physiology**

1. A 14 year old girl was found to have testes rather than ovaries in the abdominal cavity, on investigation. Further examination revealed that she had epididymis, vas deferens and seminal vesicles, but her external genitalia were female in appearance.
a. What is your most probable diagnosis?
b. Explain one possible cause for the above condition.
c. With the help of a diagram, outline the summary of normal sex determination, differentiation and development in males.
2. Give physiological basis for the following:
a. Lactation amenorrhea

**Renal physiology**

1. A medical student meets with a road traffic accident. When his friends brought him to the hospital they noticed that he was feeling intensely thirsty, his respiration was rapid and skin was cool and pale. His BP was 80/50 mmHg. He was given intravenous fluid.
a. What happens to the GFR in this patient and give the basis for the change.
b. Describe the compensatory mechanisms initiated by the juxtaglomerular apparatus in this patient.
c. With reason, comment on the urine output of the above patient.
2. Give physiological basis for the following:
**a**. Ability to concentrate urine is as high as 5000 mosm/kg in certain desert rodents

Pre-COPT (blocks 1 & 2) and post-COPT block (blocks 3& 4) essay exam scores of the experimental group of students were compared with each other using ANOVA. Post-COPT essay exam scores of the experimental group of students were compared with that of the control group using ANOVA (Repeated measures). Feedback regarding COPT was taken from the students by giving them a feedback form containing nine items. Students were asked to indicate their response by putting a tick mark in the appropriate column marked strongly agree, agree, disagree, strongly disagree and uncertain.

## Results

The performance of March 2003, September 2003 and March 2004 batches of students (experimental groups) in the pre-COPT blocks (block 1& 2) were compared with their performance in the post-COPT blocks (block 3 & 4). The results are shown in Table 2. Their scores were found to be higher in post-COPT blocks compared to pre-COPT blocks. The results of comparison of performance of experimental groups with the control group of students in the post-COPT blocks are shown in Table 3. It was observed that, the experimental group of students performed better compared to the control group.

**Table 2:** Mean (± SD) pre and post-COPT scores (out of a total of 50) of the experimental and control groups.

**Batches**	**Pre-COPT (Mean ± SD)**	**Post-COPT (Mean ± SD)**	**P-value**
March 2003	32.0 (10.3)	36.14 (8.51)	<0.001
September 2003	27.48 (9.65)	37.69 (7.68)	<0.001
March 2004	27.83 (9.14)	29.47 (8.80)	<0.001
September 2004		32.11 (9.27)	

P value significant at 0.05 level

**Table 3:** Comparison of performance of experimental group with the control group of students in the post-COPT blocks (The means of the difference from the control group).

**Batches**	**In comparison with**	**Mean (± SEM)**	**P-value**
March 2003	September 2003	1.48 (0.61)	0.04
	March 2004	5.41 (0.61)	<0.001
	September 2004	1.61 (0.61)	0.05
September 2003	March 2004	3.93 (0.62)	<0.001
	September 2004	0.13 (0.61)	1.00
March 2004	September 2004	3.79 (0.61)	<0.001

• P value significant at 0.05 level

Feedback from the students indicated in general that, COPT facilitated their learning. The results are shown in Table 4. Students felt that CTQ stimulated their thinking and improved their reasoning skills. It was also reported that they preferred this type of teaching to didactic lectures. A few students were found to be unsatisfied with this type of teaching. Feedback also indicated that COPT was well accepted and was more preferable compared to didactic lectures. Students' felt that it provides motivation for them to study physiology and that CTQ helped them to improve their reasoning skills.

**Table 4:** Students' Feedback on COPT

***Items***	***Strongly agree***	***Agree***	***Disagree***	***Strongly Disagree***	***Uncertain***
1. It motivates me to learn physiology	99 (34.38%)	175 (60.76%)	6 (2.08%)	4 (1.39%)	4 (1.39%)
2. It promotes better understanding of the subject matter	124 (43.06)	148 (51.39%)	6 (2.08%)	4 (1.39%)	3 (1.04%)
3. It helps to gain an in-depth knowledge about the subject	158 (54.86)	107 (37.15%)	4 (1.39%)	3 (1.04%)	8 (2.78%)
4. CTQ help to reduce my misconceptions about the topic	146 (50.69%)	127 (44.10%)	11 (3.82%)	3 (1.04%)	2 (0.69%)
5. CTQ stimulate my thinking	166 (57.64%)	99 (34.38%)	3 (1.04%)	4 (1.39%)	6 (2.08%)
6. CTQ improve my reasoning skills	168 (58.33%)	105 (36.46%)	4 (1.39%)	3 (1.04%)	7 (2.43%)
7. This type of teaching helps me to relate physiological principles to real life situations	151 (52.43%)	125 (43.40%)	6 (2.08%)	4 (1.39%)	2 (0.69%)
8. I feel CTQ and case studies should be included in physiology curriculum	165 (57.29%)	104 (36.11%)	6 (2.08%)	4 (1.39%)	9 (3.13%)
9. I prefer this type of teaching to didactic lectures	167 (57.99%)	96 (33.33%)	7 (2.43%)	2 (0.69%)	26 (9.03%)

## Discussion

In the present study, all the three batches of experimental group of students' performance in the essay paper were found to be significantly higher in the post-COPT blocks. Compared to the control group, experimental group of students performed better in the post-COPT blocks. Assessments that require only factual recall are notoriously unreliable indicators of real learning [8], and if assessment is to be used to ensure learning, more complex approaches are needed. Also, the assessment pattern should match with teaching pattern. The present study indicates that, the experimental group of students were able to think better and also apply theoretical knowledge in diagnosing the disorders as in clinical case studies, as evidenced by their performance in the essay paper. This could be because they were trained in answering clinically oriented questions through COPT. Whereas, the control group of students were not trained in such a way. In one of our earlier studies [9], we have reported that, analysis of mean percentage scores for recall questions and critical thinking questions in two exams, one before the incorporation of COPT (Exam 1) and one after the incorporation (Exam 2) showed a significant increase in the mean percentage score for Exam 2 (from 33 to 38%; P < 0.0001).

McParland et al [10], reported improved examination performance of undergraduate psychiatry students after the incorporation of problem based curriculum. Issac et al [11], reported that, students who followed Clinically Oriented Anatomy Teaching (COAT) fared better than those who were taught using traditional methods. Our educational approach is also similar to that of Dolmans et al [12], who suggested that basic science concepts should be presented in the context of a clinical problem, to encourage integration of knowledge.

The use of case studies holds great promise as a pedagogical technique for teaching. Faculty use case studies in their curriculum to teach content, involve students with real life data or provide opportunities for students to put themselves in the decision maker's shoes. Cases add meaning by providing students with the opportunity to see theory in practice. In COPT paradigm of teaching, students are expected to apply basic information through analysis of situations and problems focusing on how that information is relevant to the practice of medicine. Studies have suggested that intrinsic motivation is created when the relevance of the subject matter is the primary driving force [13]. This was made clear to the students when they had to work through the case studies and CTQ.

## Conclusion

In the present study, COPT was found to be a useful adjunct to didactic lectures in teaching physiology. The study supports the fact that assessment and teaching patterns should fall in line with each other as proved by the better performance of the experimental group of students who were trained to answer clinically oriented questions compared to the control group who were not trained so. COPT was also found to be a useful adjunct to didactic lectures in teaching physiology. COPT was well received by the students. They were encouraged to realize the importance of physiology in medicine and COPT served as a stimulus for their critical-thinking insights.

## Limitations of the present study

COPT could not be incorporated in all the four blocks due to time constraints. The questions in the essay examination could not be made uniform for all the batches as the commencement time of the course for each batch is different. The content covered by the examinations were different which might also have influenced the results.

## Competing interests

The authors declare that they have no competing interests.

## Authors' contributions

RA conceived the study, was involved in the data entry and wrote the first draft of the manuscript. KR was the key person in giving permission to conduct the study. He also reviewed the manuscript and contributed to the final version of the manuscript. AK helped in finding out the appropriate statistical methods for the data analyses and also in the interpretation of the results. All authors read and approved the final manuscript.

## Pre-publication history

The pre-publication history for this paper can be accessed here:


